# Salivary Oxidative Stress, Total Protein, Iron and pH in Children with β-Thalassemia Major and their Correlation with Dental Caries

**DOI:** 10.30476/DENTJODS.2021.90070.1464

**Published:** 2022-09

**Authors:** Ali Amin Akbarnejad, Soleiman Mahjoub, Ahmad Tamaddoni, Jila Masrour-Roudsari, Seyed Ali Seyedmajidi, Maryam Ghasempour

**Affiliations:** 1 Student Research Committee, Babol University of Medical Sciences, Babol, Iran; 2 Dept. of Clinical Biochemistry, School of Medicine, Cellular and Molecular Biology Research Center, Health Research Institute, Babol University of Medical Sciences, Babol, Iran; 3 Dept. of Pediatric Hematology and Oncology, Health Research Institute, Babol University of Medical Sciences, Babol, Iran; 4 Infectious Diseases and Tropical Medicine Research Center, Health Research Institute, Babol University of Medical Sciences, Babol, Iran; 5 Dental Materials Research Center, Health Research Institute, Babol University of Medical Sciences, Babol, Iran; 6 Dept. of Pediatric Dentistry, School of Dentistry, Oral Health Research Center, Health Research Institute, Babol University of Medical Sciences, Babol, Iran

**Keywords:** Thalassemia Major, Hematological Disease, Dental Caries, Child, Saliva

## Abstract

**Statement of the Problem:**

Iron overload in β-thalassemia major leads to oxidative damage to tissues, which may have an important role in the onset and progression of oral diseases.

**Purpose:**

The aim of this study was to evaluate the salivary oxidative stress indicators, total protein, iron, and pH in children with β-thalassemia major and their relationship with the status of dental caries in comparison with healthy children.

**Materials and Method:**

In this case-control study, 68 β-thalassemia major and healthy children, who were age- and sex matched, were selected. Two mililiters of saliva was collected from each child. The pH was measured using pH meter paper. Thiobarbituric acid reactive substances (TBARS) as salivary lipid peroxidation index, total antioxidant capacity (TAC), total protein, and iron were measured by spectrophotometry. Data were analyzed by SPSS ver. 22 software with Pearson and independent samples t-test.

**Results:**

TBARS, TAC, iron and dmft index in the β-thalassemia major group were significantly higher and pH was significantly lower than the control group (*p*< 0.001). The total protein difference between the two groups was not significant (*p*= 0.081).

**Conclusion:**

Considering the higher salivary TBARS in the β-thalassemia major group, oxidative stress can be considered as a risk factor for dental caries in children with β-thalassemia major. Prescription of antioxidant supplements especially natural antioxidants in the diet of children with β-thalassemia major is recommended to reduce oxidative stress.

## Introduction

β-thalassemia major is one of the most common inherited blood diseases caused by a defect in the gene, which is responsible for making the mature hemoglobin [ 1 ]. According to WHO reports, 5% of the world's population has various disorders in the alpha or beta chain of hemoglobin. This disease is more prevalent in Mediterranean coastal countries, Asia, and the South Pacific [ 1 ].

Some of the problems of these patients are related to the blood transfusion they receive; the number and the frequency of these problems depend on the patient's condition [ 2 ]. Frequent blood transfusion to compensate for anemia increases their excess load and consequently leads to oxidative stress which through the free radical production, causes the peroxidation of lipids and proteins [ 2 ]. Malondialdehyde (MDA) is one of the end products of lipid peroxidation that has an important pathophysiological effect [ 2 - 3 ]. The most common problem in thalassemia patients is oxidative stress due to iron overload because of frequent blood transfusions, which can lead to growth lag, delayed sexual development, and eventually involvement of the liver, heart, and endocrine system [ 4 ].

The β-thalassemia major is a prevalent disease in northern Iran and many studies have shown that the rate of dental caries in children suffered from thalassemia major is higher than healthy ones [ 1 , 5 - 6 ]. Though dental caries is a multifactorial infectious disease, the principal cause of higher rates of dental caries in thalassemia patients is still unclear [ 2 , 6 - 8 ]. In a study conducted in 2019 on thalassemia major patients aged 3 to 17 years old, the frequency and severity of dental caries in deciduous and permanent teeth were 2 and 3 times higher than healthy individuals, respectively [ 1 ].

Early childhood caries (ECC) is the most prevalent childhood disease with a 5-fold prevalence of asthma [ 2 ]. Microbial, immunological, behavioral and environ-mental factors play role in its development. This progressive disease affects infants and young children and since it can lead to pain, it might affect the quality of life and general health [ 2 ]. 

Saliva can be the first defensive line against oxidative stress created by free radicals. According to the results of the Mahjoub *et al.* [ 9 ] and Hedge *et al.* [ 10 ] studies, the total antioxidant capacity (TAC) of saliva increased in children with caries due to compensatory mechanisms on oxidative stress.

Until now, limited studies have been performed to compare the β-thalassemia major children’s salivary ox-idative stress indices. This study aimed to compare salivary thiobarbituric acid reactive substances (TBARS), TAC, total protein, iron and pH of β-thalassemia major children with healthy ones and their relationship to dental caries status.

## Materials and Method

This case-control study was conducted on 68 (34 β-thalassemia major and 34 healthy) children aged 3-6 years old. The study was performed after being approved by the Ethics Committee in the research of Babol University of Medical Sciences (#9031442) and the Welfare Organization; a consent form was signed by the children's parents. The inclusion criteria were consisting of the 3-6 years old thalassemia major children who had referred to Amirkola Thalassemia Center (Babol, Iran) and Taleghani Children's Hospital (Gorgan, Iran). Heal-thy age- and sex-matched cases were selected from kindergartens of same towns (Babol and Gorgan). After clinical examination by a physician, those who were involved with other thalassemia, anemia, cardiovascular, infectious, inflammatory, hepatic, and other chronic diseases were excluded. They should not have consumed any supplementation, antibiotics, multivitamins or undertaken fluoride therapy during the last 2 months. Then dental examination was performed by a trained dental student and dmft index was recorded according to WHO definition by using a dental mirror in normal and suitable room light condition [ 11 - 12 ]. Then 2 ml of unstimulated whole saliva was collected from children according to standard conditions. The children had sat on a normal chair in a quiet environment and had not eaten or brushed their teeth for at least one hour before sampling. The sampling was performed at about 9-10 am. Samples containing food particles and sputum were excluded from the study [ 13 - 14 ]. 

The samples of each child were collected and coded in a sterile, disposable laboratory container. The pH of sample was measured at the sampling site using pH meter paper (Merck, Germany) with an accuracy of 0.5 units. The collected samples were transferred to a biochemistry research laboratory using containers containing ice and stored in the freezer at -20°C until the tests were performed. After collecting all samples, the required reagents and chemical solutions were made. All samples were centrifuged (Universal 32R, Germany) at 4000 rpm and 4°C for 10 minutes after reaching laboratory temperature, and the clear supernatant was used to measure TBARS, TAC, total protein, and iron.

Tetra ethoxy propane (1,1,3,3-tetra ethoxy propane), standard albumin, trichloroacetic acid, thiobarbituric acid (TBA), hydrochloric acid, ethanol, pH meter paper (all of them Merck, Germany) and deionized water were used in this study. The spectrophotometer (JENWAY-6505-UV-Vis, UK) was used for spectrophotometry.

### Measurement of TBARS concentration as an indicator of lipid peroxidation

The basis of most spectrophotometric methods is the reaction of one molecule MDA with two molecules TBA and removal of two molecules water, which leads to the formation of a complex with maximum absorption at 535nm. Using standard sample absorption, the standard curve was drawn and based on that, the TBARS concentration of the samples was obtained [ 15 ].

### Measurement of TAC by ferric reducing antioxidant power (FRAP) method

The basis of the FRAP test is on the reduction of ferric ions to ferrous under acidic pH conditions (due to the presence of antioxidants) and production of ferric tripyridyltriazine blue colored complex that has maximum light absorption at 593nm. The linear standard curve was prepared using the serial concentrations of standard solutions of ferrous sulfate and the FRAP index of the samples were calculated and reported in micromoles per liter [ 16 ].

### Measurement of total protein by Biuret method

In this method, used to measure total protein, cupric ions in reaction with proteins’ carbonyl oxygen and nitrogen amide groups form a purple-blue complex in alkaline conditions. The color intensity is proportional to the amount of protein in the sample, which is measured at 560-520 nm [ 17 ].

### Measurement of salivary iron by Ferene-Endpoint method

In the acidic condition, transferrin bound iron is released as Fe3+ and converted to Fe2+ by reducing agents. These ions form a blue complex with Ferene, which that can be measured at a wavelength of 620-578nm. The amount of adsorption is directly related to the concentration of iron [ 18 ].

### Statistical analysis

Laboratory data and demographic information obtained from the checklist were analyzed by SPSS (Statistical Package for the Social Sciences) software ver. 22 and Pearson correlation test, Mann-Whitney test, and independent samples t-test were used. In this study, the first type error α=0.05, confidence interval=95% and *p*< 0.05 were considered significant.

## Results

The present study was performed on 68 children aged 3-6 years old (included 34 β-thalassemia major and 34 healthy children). The ratio of girls to boys was the same in both groups. In addition, the mean age of the case group was 5.05±0.98 and the control group was 5.29±0.79, which did not show a significant difference (*p*= 0.37). 

The mean values ​​and standard deviation of the studied variables in case and control groups are shown in Table 1. The results showed that the mean values ​​of TBARS, TAC, iron and dmft index in the β-thalassemia major group were significantly higher than the control group (*p*< 0.001). While the mean values ​​of salivary total protein were not significantly different between the two groups (*p*= 0.081). In addition, the pH of the children in the case group was significantly lower compared to the control group. In other words, the salivary acidity of β-thalassemia major children was higher than the control group (*p*= 0.001). The correlation between dmft and measured salivary variables of β-thalassemia major children are given in Table 2. The results showed that there was a significant positive correlation between TB-ARS, TAC, total protein and iron with dmft. While no significant, relationship was found between dmft and pH. Based on the results, there is a significant positive correlation between iron and TBARS as an indicator of lipid peroxidation in the case group, which indicates oxidative stress, as well as total antioxidant capacity (Table 3).

**Table 1:** Mean and standard deviation of salivary thiobarbituric acid reactive substances (TBARS), total antioxidant capacity (TAC), iron, total protein, and pH in children with β-thalassemia major and healthy ones

Variable	Group	N	Mean	Std. Deviation	*p* Value
TBARS (µmol/l)	Case	34	0.28	0.08	<0.001
	Control	34	0.14	0.07	
TAC (mmol/l)	Case	34	1.13	0.46	<0.001
	Control	34	0.36	0.16	
Iron (µg/dl)	Case	34	1175.67	298.38	<0.001
	Control	34	368.73	128.68	
Total Protein (mg/dl)	Case	34	0.25	0.26	0.18
	Control	34	0.15	0.14	
pH	Case	34	6.57	0.84	0.001
	Control	34	7.25	0.67	
dmft	Case	34	7.00	3.93	0.03
	Control	34	4.85	3.21	

**Table 2:** Correlation between dmft and salivary thiobarbituric acid reactive substances (TBARS), total antioxidant capacity (TAC), iron, total protein and pH in children with β-thalassemia major

	Variables	*p* Value	Correlation coefficient
dmft	TBARS (µmol/l)	0.004	+0.278
	TAC (mmol/l)	0.019	+0.228
	Total Protein (mg/dl)	0.011	+0.245
	Iron (µg/dl)	0.001	+0.307
	pH	0.062	-0.182

**Table 3:** Correlation between salivary iron and thiobarbituric acid reactive substances (TBARS) and total antioxidant capacity (TAC) in children with β-thalassemia major

	Variables	*p* Value	Correlation coefficient
Iron (µg/dl)	TBARS(µmol/l)	< 0.001	+0.533
	TAC(mmol/l)	< 0.001	+0.638

## Discussion

This case-control study was performed on β-thalassemia major and healthy children to obtain more information about the correlation of oxidative stress and dental caries. Salivary TBARS, TAC, iron, and total protein of β-thalassemia major children were determined and compared with healthy children. Except total protein, other parameters were significantly higher in β-thalassemia major children. Saliva is a biological environment that plays an important role in the oral ecosystem and the prevention of tooth decay [ 19 ]. Analysis of human salivary compounds is necessary to understand the protective properties of this biological fluid better. The buffering ability of saliva is effective in regulating pH and dental remineralization [ 19 ]. 

Oxidative stress is the most common problem in thalassemia patients due to iron overload because of frequent blood transfusions [ 20 ]. Iron can convert molecular oxygen to radical active species through the Fenton reaction, resulting in oxidative damage. Increased iron increases the transferrin-bound iron in the plasma and non-transferrin plasma-binding iron (NTBI). Toxicity of NTBI is much higher than transferrin-bound iron, as its ability to stimulate the formation of hydroxyl radicals, which causes the peroxidation of membrane lipids and proteins [ 20 ]. In thalassemia patients, there is evidence of the presence of low molecular weight iron in the serum and intracellular storage of it. This iron damages the membranes of cells and intracellular organs, especially in overloaded organs such as the liver, pituitary, pancreas, and heart [ 4 ]. Increasing iron, especially free divalent iron, has a direct effect on increasing lipid peroxidation and its specific metabolite, MDA [ 20 ].

Based on the findings of the present study, due to the higher rate of salivary TBARS and dental caries in β-thalassemia major group in comparison with the control group and the positive correlation between them, oxidative stress in these patients also can be considered as a risk factor for ECC in children. Das *et al.* [ 21 ] measured the TBARS in the erythrocytes of 8 thalassemia patients and 6 healthy ones (aged 4-16 years old). Şimşek Orhon *et al.* [ 22 ] also measured plasma MDA levels in 11 thalassemia major patients and 10 healthy individuals with a mean age of 7 years old. The results of these two studies showed that the rate of lipid peroxidation in these patients is higher than healthy ones, which is in agreement with the results of our study on children aged 3-6 years old and the results yielded by the study of Kassab-Chekir *et al.* [ 23 ] on β-thalassemia children.

Due to the diversity of enzymatic and non-enzymatic antioxidants, their separate study is both difficult and costly, so in this study, we used the FRAP assay as a valid method to determine the TAC. Higher levels of total salivary antioxidants in the β-thalassemia major group, which also has a higher caries rate, indicate that the levels of antioxidants change in response to infection and disease. It seems that the higher oxidative damage caused by more caries in these patients is a factor to increase the total antioxidant capacity to counteract the oxidative stress increase. In the study of Jouda *et al.* [ 4 ], which was performed on 4-30 years old individuals, this factor was higher in the saliva of thalassemia patients than in the control group.

In the present study, it was shown that the concentration of salivary iron in the case group is higher than in the control group. This high concentration of iron is a sign of iron overload in β-thalassemia major children, which leads to lipid and protein compounds peroxidation increased. Besides, a positive correlation was also observed between salivary iron concentrations and TBAR-SS in β-thalassemia major children. These findings are in agreement with the results of some other studies [ 24 - 25 ].

Increasing in salivary total protein could be considered as a risk factor for dental caries due to its effect on salivary flow rate and on buffering capacity [ 26 ]. According to the results of the present study, conforming to the results of the study conducted by Ghasempour *et al.* [ 8 ], the amount of salivary total protein in β-thalassemia major children was not different from the control group, which cannot be considered as a factor of higher dmft in β-thalassemia major children. Unfortunately, there are very few studies on this factor in thalassemia patients, but contradictory results have been reported in healthy individuals. Numerous biological systems involved in tissue regeneration, have also been introduced in saliva. One of them is the peroxidation complex system [ 27 ]. The main components of this system are various compounds of lactoperoxidase and myeloperoxidase, which are secreted by the salivary glands and polymorphonuclear neutrophils, respectively. One of the main roles of salivary peroxidases is to control oral bacteria that cause caries [ 27 ]. On the other hand, increasing the protein content of saliva has a direct effect on the viscosity of saliva, which may play a role for dental caries [ 28 ].

In the present study, the pH of saliva was lower in the case group in comparison with the control group. According to Luglie *et al.*'s [ 29 ] study, due to the changes that occur in the saliva composition of patients with β-thalassemia major, the lower pH of their saliva can be attributed to the lower salivary urea levels. Decreased salivary urea plays a significant role in lowering plaque pH, as hydrolysis of urea can maintain plaque pH at normal levels. However, according to Bhat *et al.*’s [ 30 ] study performed on 6-16 years old individuals, there was no relationship between the pH of saliva of thalassemc and normal individuals.

The results of our study did not show a significant relationship between the pH of saliva and tooth decay index. In Preethi *et al.*'s [ 27 ] study, in children with ECC, the salivary pH levels were not lower than that in caries-free children. It can be assumed that other factors such as microbial flora, diet, and oral hygiene have stronger effects on pH at the beginning of the caries process, which is a multifactorial disease.

Children with β-thalassemia major disorder suffer from iron overload and oxidative damage due to frequent blood transfusions, which play an important role in the onset and progression of oral and dental diseases in them. Due to the many systemic problems that β-thalassemia major children and their parents are involved with, recognizing the factors affecting dental caries can be an effective step in preventing them.

Finding children with β-thalassemia major according to exclusion criteria and collection of saliva samples were major problems in this study. It can be noted that although in these patients, iron is removed; this condition indicates the inadequacy of chelation therapy in them. Prescription of antioxidant supplements in β-thalassemia major children’s diet in order to reduce oxidative stress and subsequently, investigation of its effects on salivary biochemical condition is suggested as a clinical trial study for future.

## Conclusion

Based on the findings of the present study, due to the higher level of TBARS, TAC, iron, and dmft index in β-thalassemia major children compared to the healthy ones, oxidative stress can also be considered as a risk factor in the incidence of caries in these children. Prevention of oral diseases in β-thalassemia major patients is very important to improve the oral health condition and consequently the quality of life, and in this regard, prescribing antioxidant supplements especially more natural antioxidants in their diet for the reduction of the oxidative stress is suggested.

## Acknowledgment

The authors thank Vice-Chancellery for Research and Technology of Babol University of Medical Sciences and Dr. Narges Beigom Mirbehbahani (Taleghani Children's Hospital, Golestan University of Medical Sciences) and Mr. Hemmat Gholinia for their assistance during this research.

## Conflict of Interest

The authors declare no potential conflicts of interest with respect to the authorship and/or publication of this paper.
